# Supplementary material for: The adaptive value of density-dependent habitat specialization and social network centrality
Source: Nat Commun. 2024 May 24;15:4423. doi: 10.1038/s41467-024-48657-8 (PMC11126670; doi:10.1038/s41467-024-48657-8)
Supplement: Supplementary file 1 — Supplementary Information [file 41467_2024_48657_MOESM1_ESM.pdf]

Supplementary Information

Supplementary Note 1: *Additional information on study area, data collection, and subjects*

We studied adult female caribou (*Rangifer tarandus*) from six herds (Buchans, Grey River, La Poile, Middle Ridge, Pot Hill, and Topsails) on the Island of Newfoundland, Canada (Figure S1: 47°44'N, 52°38'W to 51°44'N, 59°28'W) between 2007–2013 (Table S1). Caribou (n = 127 individuals) were immobilized by a dart fired from a helicopter and outfitted with global positioning system (GPS) collars (Lotek Wireless Inc., Newmarket, ON, Canada, GPS4400M collars, 1,250 g). Collars were deployed on adult female caribou for one to three years, but collars were often re-deployed on the same individuals for up to seven years. Consistent with most ungulate monitoring practices in North America, including elk (Harris *et al.* 2010) and caribou (Virgl *et al.* 2017), only adult female caribou were monitored using GPS collars during the period of our study. In ungulates, adult females are considered the most important demographic class (Festa-Bianchet *et al.* 1998; Gaillard *et al.* 2000) for population dynamics. Furthermore, where adult females have little variance (elasticity) in their survival rates, offspring survival – or annual reproductive success – is highly variable. Thus, because variance in offspring survival it is an important and promising demographic class from which to detect effects. For these reasons, most management agencies (including the Newfoundland and Labrador Wildlife Division) deploy collars on adult females as the objectives of these agencies is to answer questions about vital rates (e.g., reproductive success) and to monitor survival of calves. Given these management objectives, in combination with the cost of deployment and maintenance of collars, adult males are not typically collared by management agencies.

Typically, males are relatively solitary from spring through to the autumn rut (Webber & Vander Wal 2021). Moreover, the male:female sex ratio for caribou in Newfoundland is skewed

towards females (e.g. ~0.8 males per female: (Mahoney & Virgl 2003), presumably due to hunting pressure against males (Ellington *et al.* 2020; Weir *et al.* 2014). While data for males was unavailable for our study, our inference is restricted to how behaviour of females affects the survival of their calves (see Main Text).

The Newfoundland and Labrador Department of Environment and Conservation captured and immobilized caribou by aerial darting from a helicopter with a mixture of carfentanil (12 mg/kg) and xylazine (0.2 mg/kg) or a mixture of ketamine (2 mg/kg) and xylazine (6 mg/kg) administered intramuscularly with a CO2-powered pistol (Palmer Cap-Chur Inc., Power Springs, Georgia). All collars were programmed to record locations every two hours, depending on herd, season, and year. Prior to analyses we screened telemetry data and removed all erroneous fixes (Bjørneraas *et al.* 2010). Animal capture and handling procedures conformed to guidelines established by the American Society of Mammalogists (Sikes & Mammalogists 2016).

Caribou ranges in Newfoundland comprise coniferous and mixed forest dominated by balsam fir (*Abies balsamea*), black spruce (*Picea mariana*), and white birch (*Betula papyrifera*), as well as bogs with stunted black spruce and tamarack (*Larix laricina*). Barren rock, lakes, and ponds are also common land features in Newfoundland. Caribou in Newfoundland have undergone drastic changes in abundance over the last 50 years, with low abundance from the 1950s to 1970s, followed by rapid growth in the 1980s and 1990s, and precipitous declines in the 2000s that persists to the present (Bastille-Rousseau *et al.* 2013a). Broad-scale changes in population abundance were consistent across herds (Figure S2).

Newfoundland has a humid continental climate with cool temperatures and high precipitation throughout the year. It is reasonable to assume that climatic variation could influence habitat or resource specialization for caribou through the availability and access to

nutritional vegetation for caribou (Schaefer *et al.* 2016). In turn, this could affect the probability of adult survival or reproductive success. However, the potential correlation between climate and demography in caribou is focused on calf survival (Bastille-Rousseau *et al.* 2016). Caribou populations in Newfoundland (and throughout much of their range) are cyclic over multiple decades (Bastille-Rousseau *et al.* 2013b; Gunn 2003) and we posit it is unlikely that rainfall or temperature affect long-term changes in population density. Population size is measured as the number of animals that have recruited into the population, i.e., not calves. Population surveys were conducted primarily between February and April (Ellington *et al.* 2020) and so our estimates of abundance (and density) exclude calves. Moreover, calf survival is ~20% regardless of extreme weather (Ellington *et al.* 2020), suggesting that even if extreme weather reduces calf survival for a single year (Bastille-Rousseau *et al.* 2016), it is unlikely that this would impact overall population density. We therefore expect it unlikely that climate change and extreme weather events affects habitat or resource specialization through population density within the time scales of our study.

Supplementary Note 2: *Supplementary information on social networks and social network randomization*

We used the *spatsoc* package (Robitaille *et al.* 2019) to generate proximity-based social networks from GPS telemetry data. As described in the main text, we applied the 'chain rule' to designate groups. Edges in each network were weighted based on the strength of association between dyads of caribou using the simple ratio index (Cairns & Schwager 1987), SRI:

$$SRI = \frac{x}{x + y_{AB}} \text{ equation (1)}$$

where x is the number of times individuals A and B were within 50 m of each other and y_{AB} is the number of simultaneous fixes from individuals A and B that were separated by >50 m (Farine & Whitehead 2015). We constructed social networks with the *igraph* package in R (Csárdi & Nepusz 2006). Nodes in our networks represented individuals and edges represented associations between individuals based on the SRI.

Although we model the relationship between social strength, habitat specialization, and fitness using Bayesian inference (see main text, Tables S6–S9), we also developed data-stream permutations to assess the potential for non-random (i.e. social associations that arise by chance as opposed to through biologically relevant processes) social structure through space and time (Farine 2017). Thus, separate from our Bayesian models, we generated null models based on GPS fixes to reduce potential for type II error typically associated with node-based permutations (Farine 2014). Following Spiegel *et al.* (2016), we re-ordered daily GPS movement trajectories for each individual while maintaining the temporal path sequence within each time block (e.g., day 1 and day 2 may be swapped). This technique is a robust network randomization procedure for GPS data because: 1) it maintains the spatial aspects of an individual's movement; and 2) by randomizing movement trajectories of individuals independent of one another, temporal

dependencies of movement are decoupled (Spiegel *et al.* 2016). We repeated this procedure 100 times for each season-by-year social network and re-calculated social strength at each iteration. We then iteratively re-ran our top mixed models and extracted the coefficient estimate for social strength as a predictor of survival (Figure S4) and the proportional similarity index (Figure S5). We considered the relationship to be non-random if the observed coefficient estimate fell outside the 95% confidence interval of the randomly generated distribution of coefficient estimates.

Supplementary Note 3: *Resource Selection and Habitat Specialization*

To determine the extent to which habitat specialization (i.e., proportional similarity index, see below) was related to individual selection of specific habitats while accounting for habitat availability, we generated resource selection functions (RSFs). For each individual, we separated data into unique season-year combinations within which used locations were regressed against randomly generated locations within the home range (Johnson 1980). RSFs produce a function that describes the relative probability of habitat selection across a landscape to quantify the habitat preference of a species. We used all GPS fixes from each individuals seasonal home-range to generate 95% MCPs to define available habitat. We then modeled RSFs using a 1:10 ratio of used to available points within the MCP home range. Given our large sample size (i.e., n = 708,248 used points; N = 7,082,471 available points) we were computationally limited in our ability to increase the ratio of used to available points (Northrup *et al.* 2022; Street *et al.* 2021). Instead of increasing the ratio of used to available points, we re-generated the set available points used in our model three times to ensure stability of coefficients.

Our goal with the RSF model was to quantify patterns of habitat selection for individuals in each of four habitat types and compare these values to measures of habitat specialization. The proportional similarity (PS_i) is a measure of interspecific dietary overlap and evenness which accounts for the amount of a resource an individual consumes relative to the population (Bolnick *et al.* 2002). Using the number of spatial relocations for a given individual in each habitat type, we estimated the proportional specialization index (PS_i):

$$PS_i = 1 - 0.5 \sum_j |p_{ij} - q_j| \text{ equation (2)}$$

Values of PS_i closer to one reflect individuals that consume resources, or in our case select habitats, in direct proportion to the population, i.e., habitat generalists, whereas values of PS_i

closer to zero reflect individuals that are habitat specialists (Zaccarelli *et al.* 2013). An important distinction between the PS_i and RSFs is that the PS_i measures specialization of a given resource relative to the population, whereas RSFs measure selection of a given habitat relative to availability of that habitat in the environment. For example, if individual A selects lichen habitat, but the population also selects lichen habitat along with other habitat types, individual A remains a relative generalist because they are no more specialized on lichen than the population. Within the context of the habitat selection literature, the original premise was that habitat generalists use habitats in proportion to their availability, i.e., no selection for nor against a given habitat (Fortin *et al.* 2008; McLoughlin *et al.* 2006). By contrast, the original premise of resource specialization-generalization within the ecological niche literature (Bolnick *et al.* 2002, 2003; Carlson *et al.* 2021) accounts for the relative relationship (e.g. proportional similarity, difference, or overlap) an individual has with other members of the sampled population. The distinction in habitat specialization between resource selection and ecological niche perspectives therefore lies in the understanding that an individual's value of PS_i reflects their propensity to consume (or select) more, or less, resources relative to the population. The relationship between the PS_i and resource selection as measured using RSFs have two outcomes. First, generalists are predicted to neither select nor avoid habitats or to have moderate selection for multiple habitats. In this scenario, the variance in selection for different habitats is relatively low. Second, strong selection for a single habitat means that, by definition, those individuals must avoid other habitat and therefore can be interpreted as specialists relative to the population. In this scenario, the variance in selection for different habitats is relatively high.

Following (Muff *et al.* 2020), we used the glmmTMB function (Brooks *et al.* 2017) to generate RSFs and extract individual measurements of selection for a given habitat type. Fixed

effect explanatory variables in our RSF included elevation and the proportion of four land-cover classes (lichen, rocky barrens, wetland, and conifer/scrub) within a 100 m buffer of each used and available location and we included each of the four land-cover classes as a random effect in the model to vary by individual-by-season-by-year. We extracted resource selection coefficients for each individual-by-season-by-year and compared them to habitat specialization measures for the same time period.

To help contextualize the relationship between PS_i and habitat selection, we illustrate the relationship between habitat specialization and habitat selection for individual caribou. We used a linear mixed effects model with a Gaussian distribution fit in the lme4 package (version 1.1.32) in R. The model included each habitat type (lichen, rocky barrens, wetland, and conifer/scrub) in an interaction with season as well as year as fixed effects and individual identity nested within herd as a random effect (Table S3). We also extracted marginal and conditional R^2 values to estimate the variance explained by the fixed effects (marginal) and random (conditional) effects (Nakagawa & Schielzeth 2013).

Habitat specialization was positively correlated with lichen and wetland selection, indicating that generalists tended to have stronger lichen and wetland selection, while habitat specialists tended to have weak selection or avoidance for the other (Table S3; Figure S5). In addition, no relationship between habitat specialization and selection for rocky barrens or conifer scrub conifer (Table S3). Together, these findings support our *a priori* expectation that habitat specialists tend to select one of wetland, rocky barrens, or conifer forests more than the others and more than lichen (Figure S6). Specifically, individuals with high fitness tend to be specialists (Figure 3 from the main text), but to obtain high fitness, these individuals specialize on alternative habitats relative to the population. Caribou select lichen throughout the year and

given the strong population-level selection for lichen and that the PS_i measures an individual's resource use proportional to the population, it is difficult for an individual caribou to be a lichen specialist. By contrast, individuals with low fitness tend to be habitat generalists (Figure 3 from the main text) and tend to have weak selection or no preference for lichen and wetland. Taken together, these results support the idea that generalists have patterns of habitat selection, which are similar to the population, i.e., select for lichen and wetland, and as a result of high competition among generalists for lichen.

Supplementary Note 4: *Additional Behavioural Reaction Norm Methods*

Behavioural reaction norms (BRNs) represent behavioral phenotypes expressed by individuals across an environmental gradient, where the BRN intercept reflects personality and the BRN slope reflects plasticity (Dingemanse *et al.* 2010). We used BRNs to evaluate repeatability and plasticity of social strength and habitat specialization as well as their relationship with fitness across a population density gradient. Models included social strength, habitat specialization, and reproductive success as co-response variables in a tri-variate Bayesian mixed model (package MCMCglmm: (Hadfield 2010)) as a function of mean-centered population density. To assess effects of individual (I) and environment (E) on our response variables we parameterized three models. First, we parameterized a global model which included all data (Table S5). Next, we separated our dataset based on individuals that experienced relatively high and relatively low density. Specifically, for the high density data subset we included the upper 75% quantile of density data and for the low density data subset we included the lower 25% quantile of density data. We then parameterized separate bivariate models at high and low density to investigate the relationship between habitat specialization and survival (Tables S6 and S7) and social strength and survival (Tables S8 and S9).

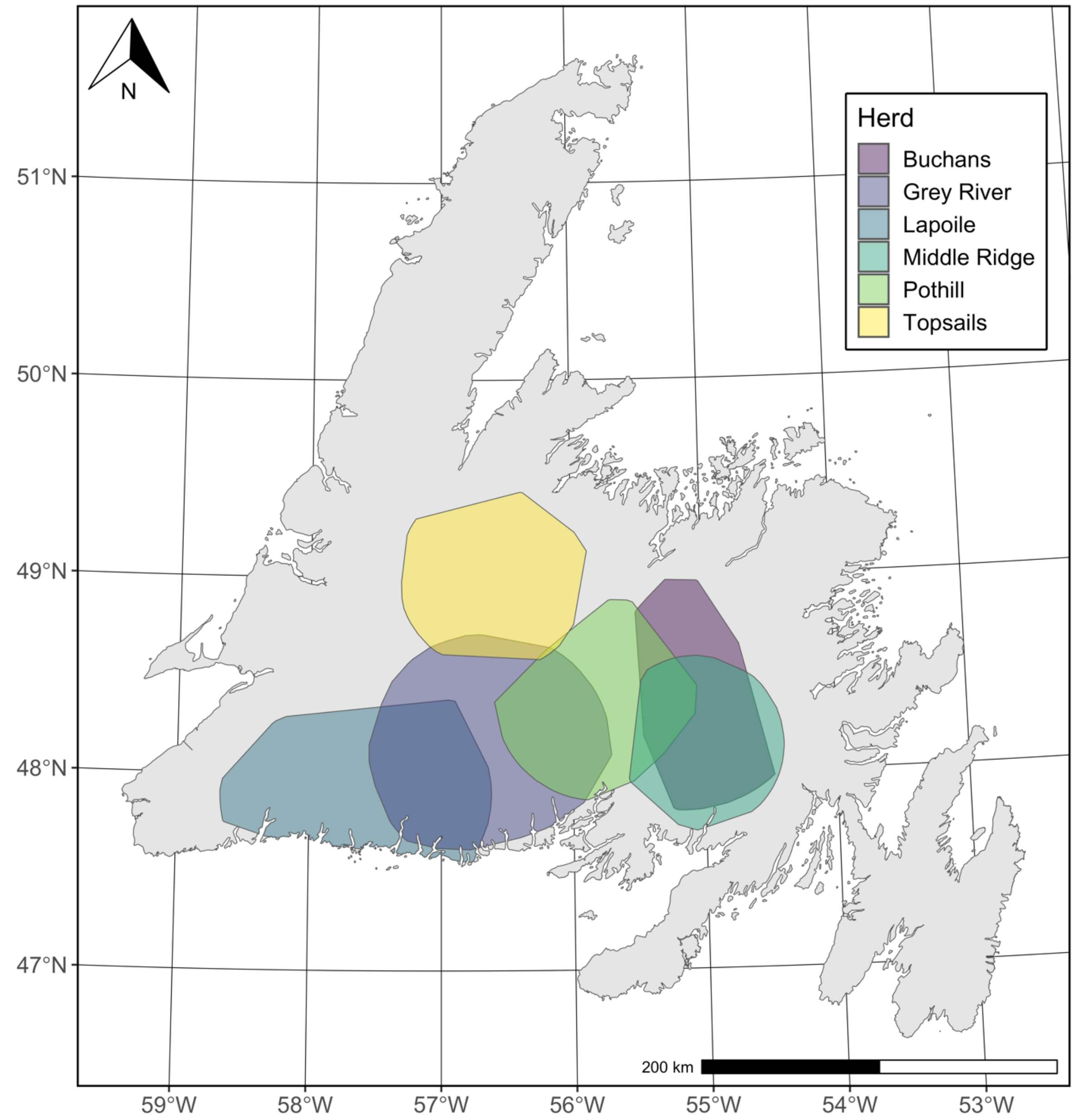

Figure S1: Map of study area including six Newfoundland caribou (*Rangifer tarandus*) herds used for data collection between 2007–2013.

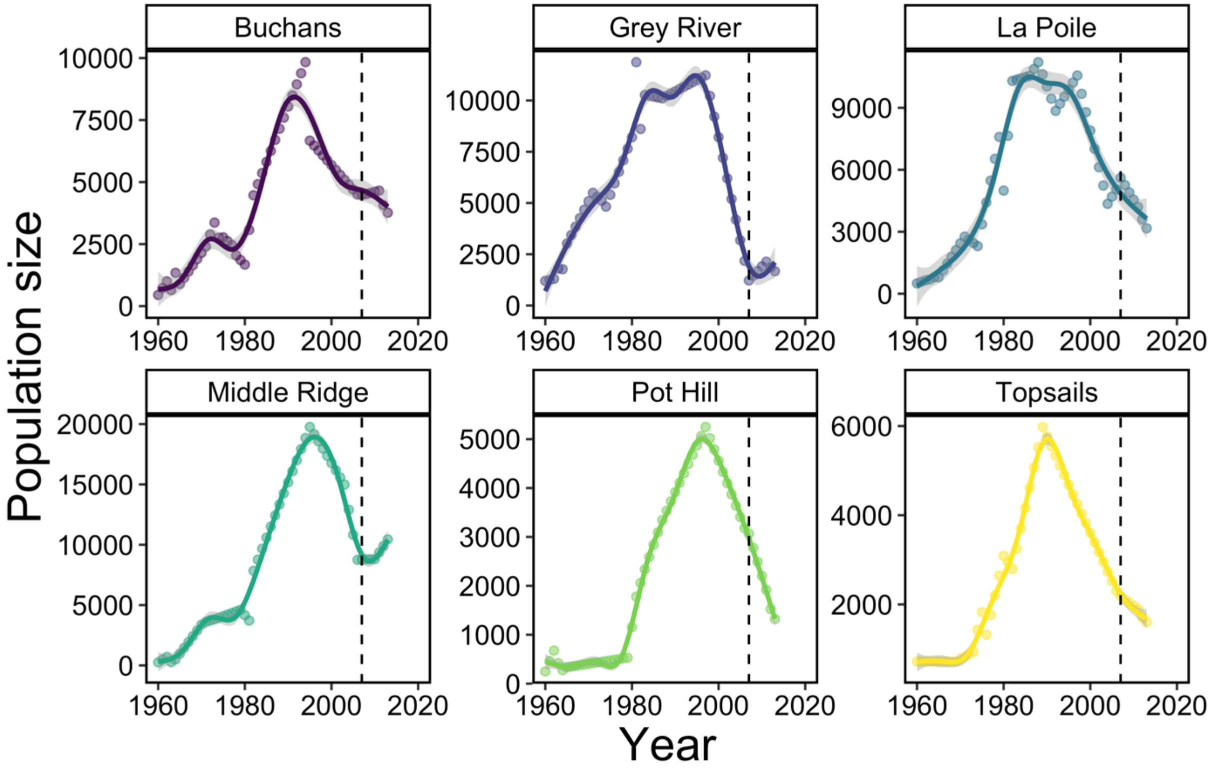

Figure S2: Herd-level changes in population size estimates between 1960 and 2013 for six caribou (*Rangifer tarandus*) herds in Newfoundland, Canada. For full details and description of population density data collection see Lewis & Mahoney (2014). Vertical dashed lines are placed at 2007, the date at which GPS collar data for this study were collected. From 2007 to 2013, Middle Ridge and Grey River herds increased in size, while Buchans, La Poile Pot Hill, and Topsails decreased in size. Note, y-axes differ for each herd for ease of interpretation.

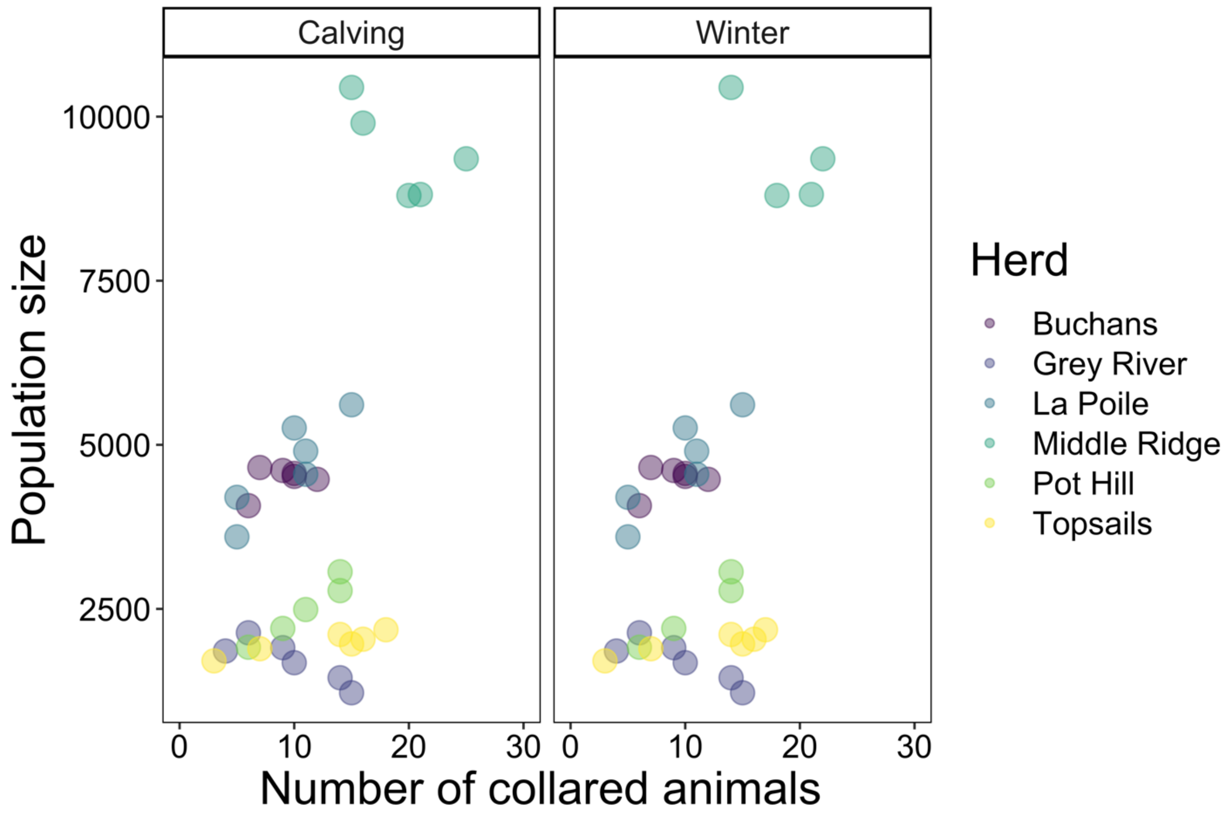

Figure S3: Herd-level differences in the number of collared animals per year as a function of population size estimates between 2007 and 2013 for six caribou (*Rangifer tarandus*) herds in Newfoundland, Canada.

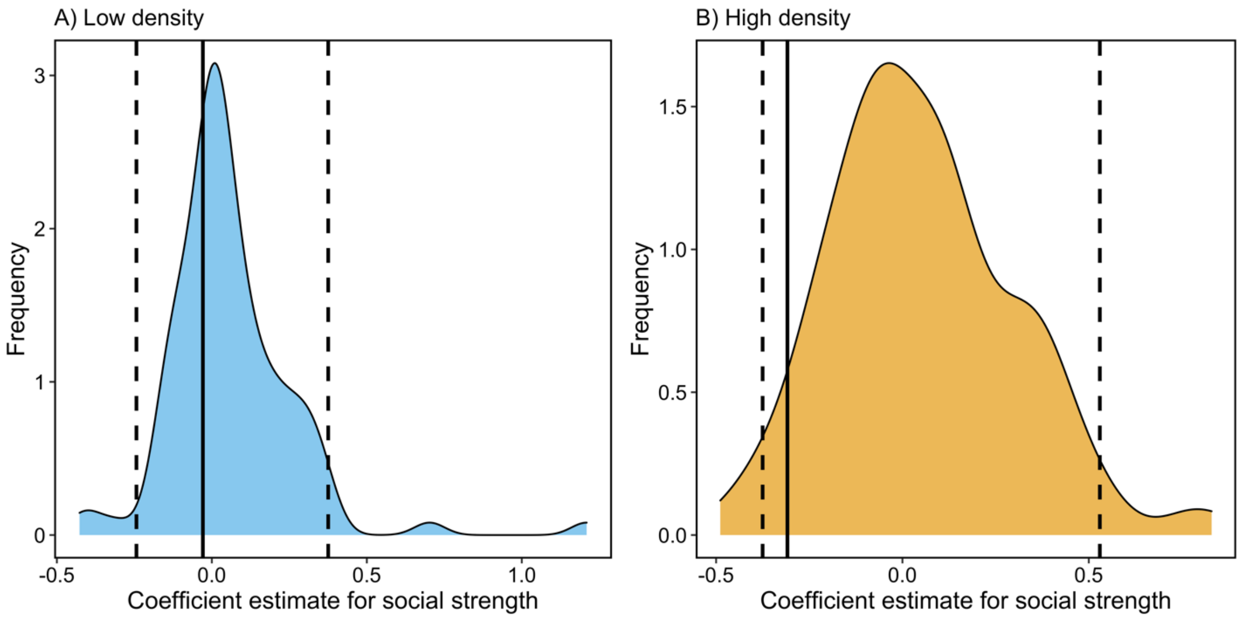

Figure S4: Comparison of observed coefficient estimates from models testing the effect of social strength on calf survival at A) low and B) high density for caribou (*Rangifer tarandus*) to the distribution of randomly generated coefficients based on network permutations. Observed coefficients are denoted by solid vertical lines and dashed lines represent 95% confidence intervals around the randomly generated distribution.

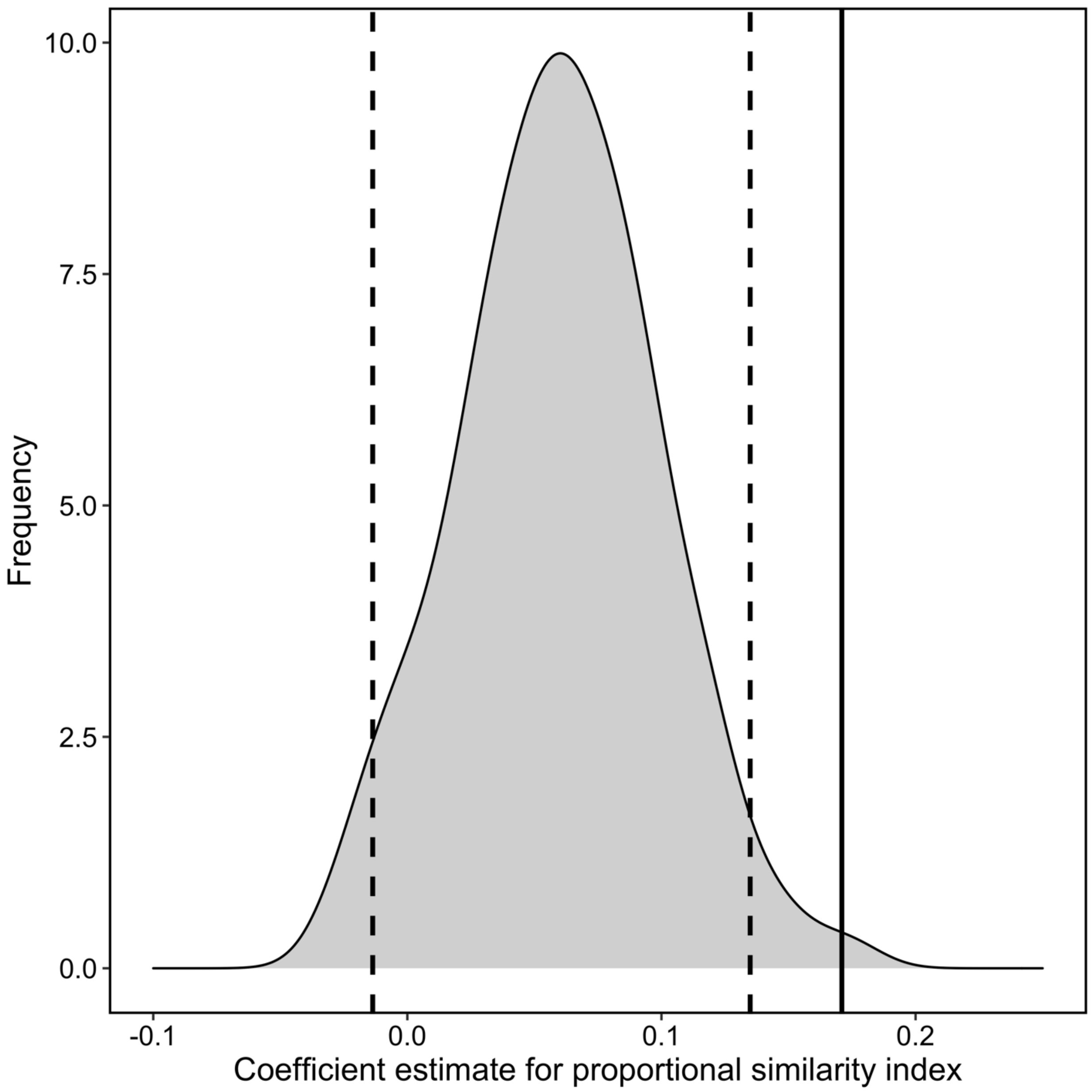

Figure S5: Comparison of observed coefficient estimate from a model testing the effect of habitat specialization (i.e., proportional similarity index) on calf survival for caribou (*Rangifer tarandus*) to the distribution of randomly generated coefficients based on network permutations. The observed coefficient is denoted by the solid vertical line and dashed lines represent 95% confidence intervals around the randomly generated distribution.

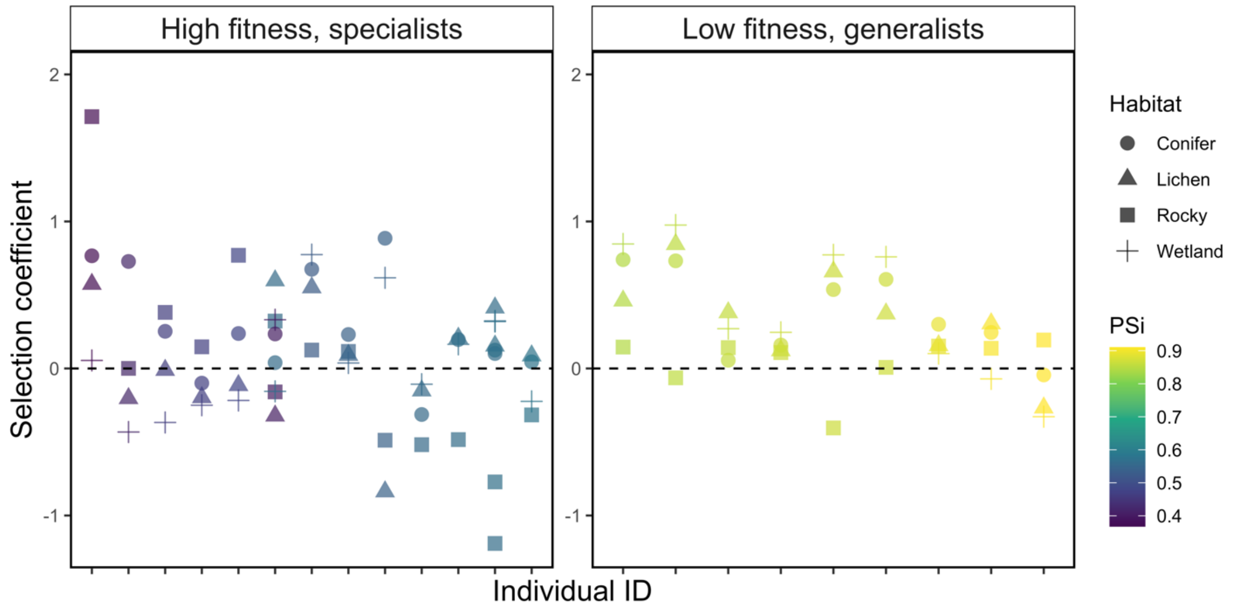

Figure S6: Comparison between habitat specialization (measured using the proportional similarity index, darker colours represent more specialized individuals and yellow colours represent more generalized individuals) and habitat selection coefficients (extracted from resource selection functions) for high (left panel) and low (right panel) fitness caribou at opposite ends of the habitat specialization-generalization continuum. Data presented here was extracted from the upper lefthand corner (i.e., specialists with high fitness at high density) and lower righthand corner (i.e., generalists with even selection and low fitness at high density) of Figure 3 in the main text. For high fitness individuals, habitat specialists tend to have uneven selection. Specialists had stronger selection for a single habitat type and either had equivocal selection or avoided other habitat types. Note high-fitness specialists occupy more data space than low-fitness generalists. In contrast to high-fitness specialists, low-fitness generalists tended to be more even and more equivocal in their selection, neither select nor avoid any habitats.

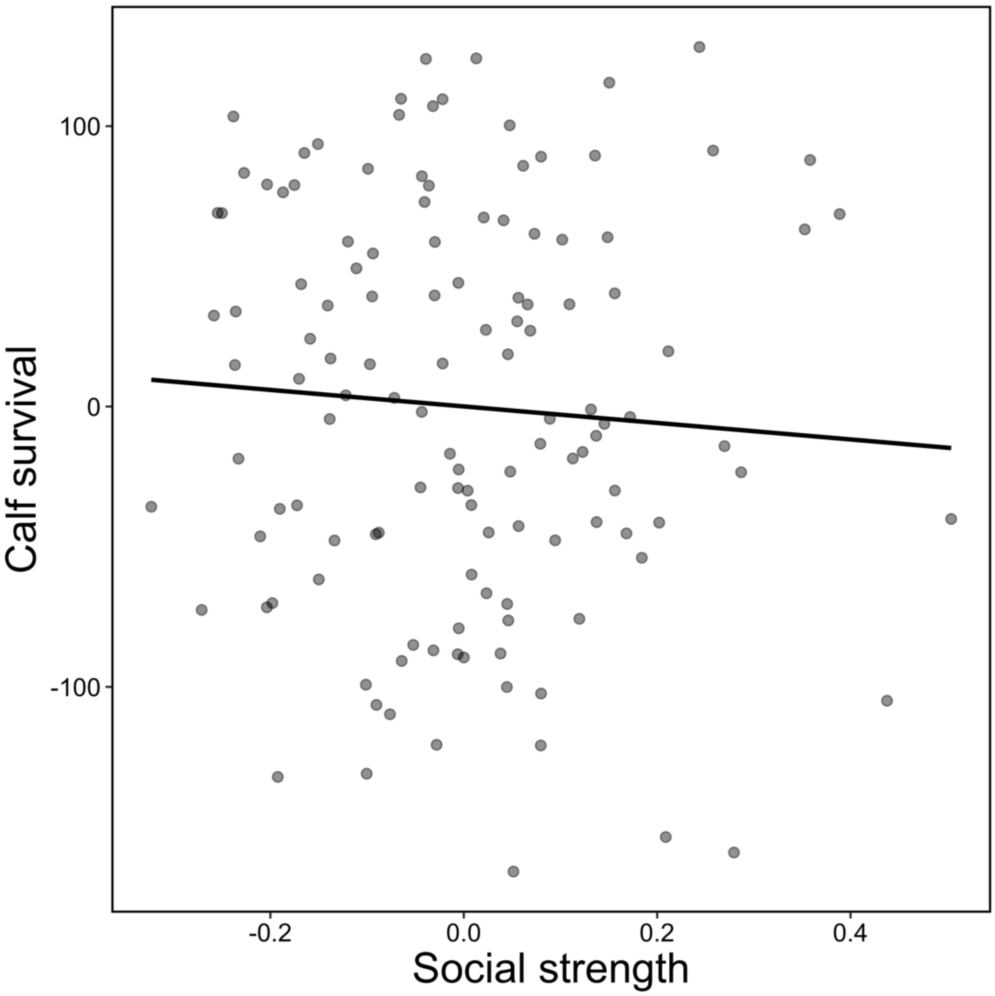

Figure S7: Phenotypic covariance between calf survival, extracted from the model as best-linear unbiased predictors, and social strength for caribou (*Rangifer tarandus*, n = 127) in Newfoundland (see Table 3 in the main text for summary of phenotypic covariance estimates).

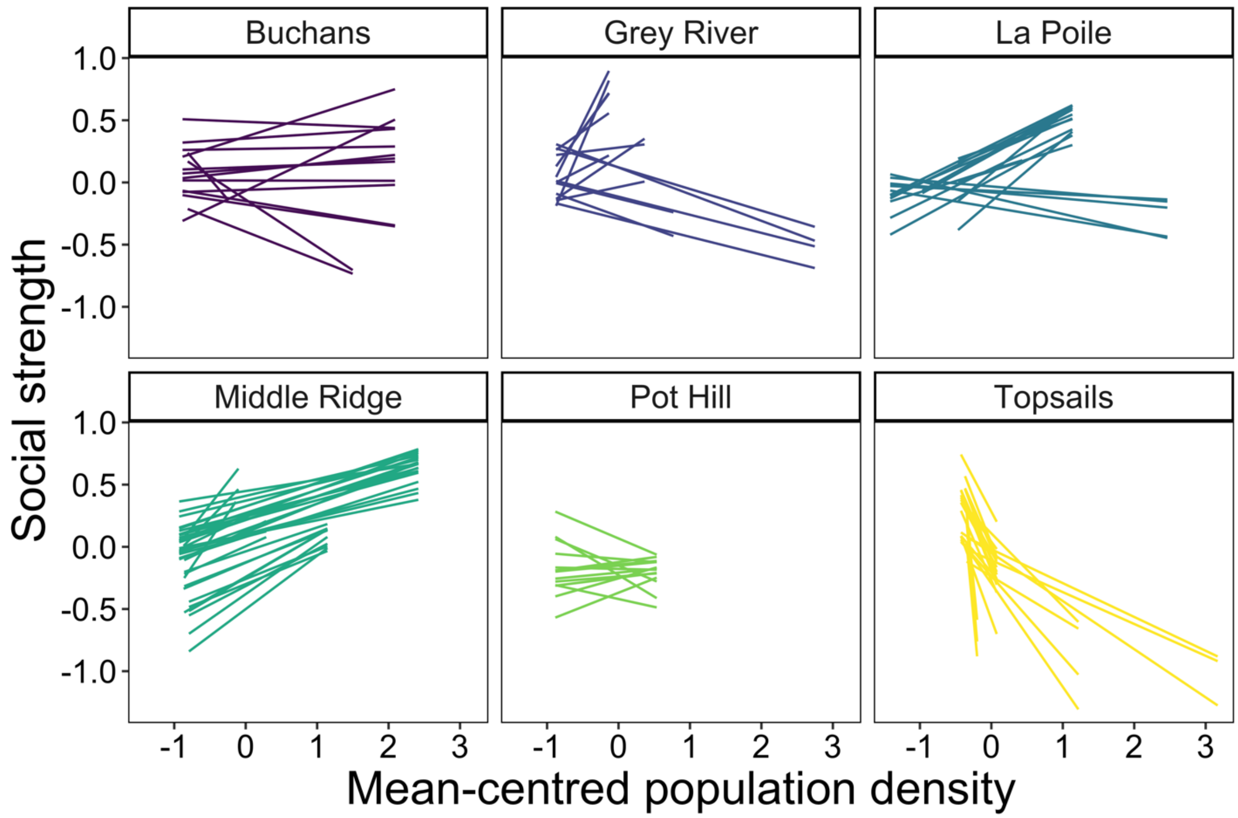

Figure S8: Behavioural reaction norms testing the relationship between social network strength and population density for caribou (*Rangifer tarandus*; n = 127) in six herds in Newfoundland. Each line represents an individual behavioural response to changes in population density and crossing of lines represents individual differences in plasticity (i.e., an individual-environment interaction).

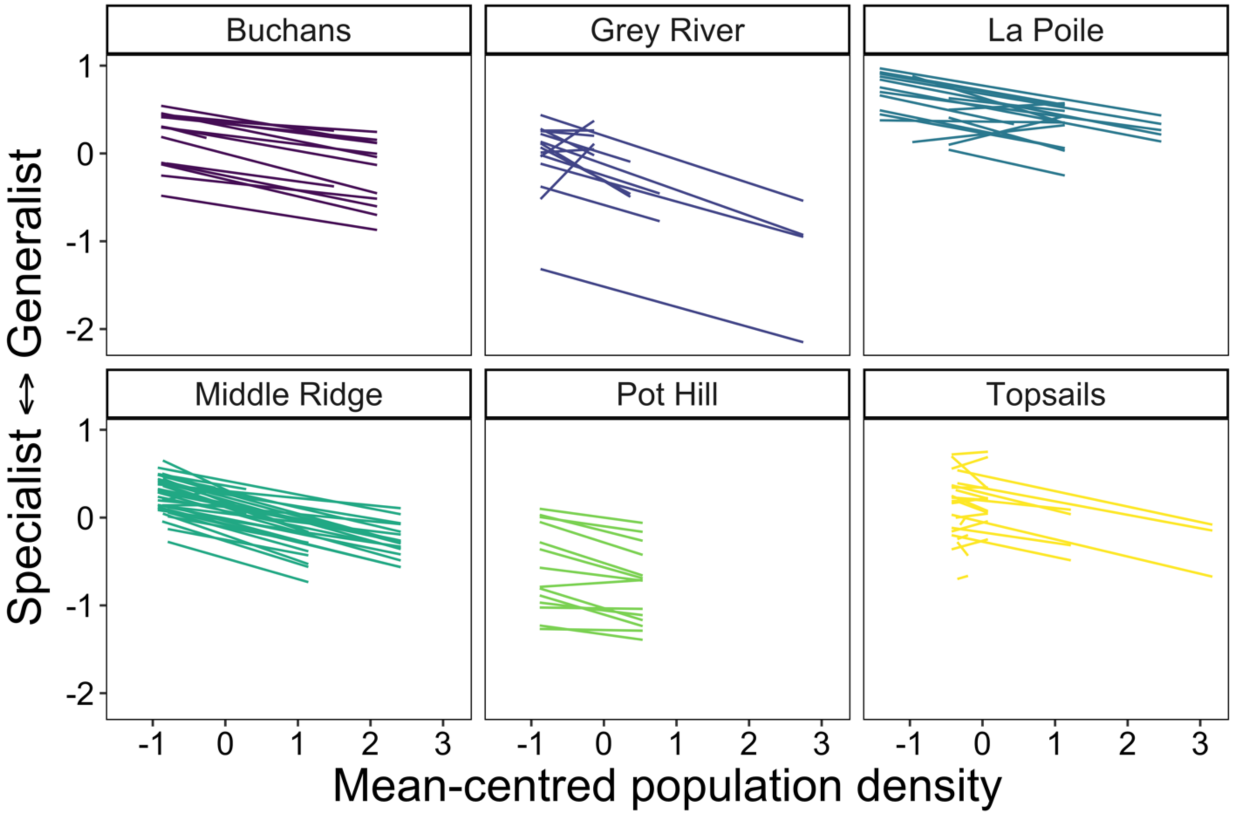

Figure S9: Behavioural reaction norms testing the relationship between habitat specialization and population density for caribou (*Rangifer tarandus*; n = 127) in six herds in Newfoundland. Each line represents an individual behavioural response to changes in population density and crossing of lines represents individual differences in plasticity (i.e., an individual-environment interaction).

Table S1: Number of individual caribou (*Rangifer tarandus*) used for social network analysis and subsequent modeling in each year-by-season combination.

Year	Calving	Winter
2007	74	73
2008	62	62
2009	78	76
2010	74	74
2011	56	53
2012	34	34
2013	15	14

Table S2: Number of GPS relocations in each of eight habitat type separated by herd for 127 caribou (*Rangifer tarandus*) in Newfoundland, Canada between 2007–2013.

	**Buchans**	**Grey River**	**La Poile**	**Middle Ridge**	**Pot Hill**	**Topsails**	**All herds**
Wetland	32,797	25,886	29,992	72,134	17,269	48,145	226,223
Lichen barrens	32,147	31,912	33,248	32,692	23,757	26,255	180,011
Conifer scrub	12,439	21,244	14,237	26,237	35,942	22,580	132,679
Conifer forest	7,414	8,414	3,624	12,571	13,271	10,881	56,175
Water/ice	5,246	6,985	4,529	11,846	4,449	7,922	40,977
Rocky barrens	6,403	3,443	5,706	7,585	1,135	4,163	28,435
Mixed-wood forest	1,699	1,505	1,673	1,005	957	4,020	10,859
Deciduous forest	97	320	1690	23	61	616	2,807
Total	98,242	99,709	94,699	164,093	96,841	124,582	681,560

Table S3: Summary of linear mixed model testing the relationship between habitat specialization and habitat selection coefficients for lichen barrens, rocky barrens, wetland, and conifer scrub as well as season and year ($R2_m = 0.16$ and $R2_c = 0.42$). Individual identity was nested within herd as a random effect in the model. P-values are two-sided, multiple comparisons were not considered, and p-values of <0.001 are noted if the estimated p-value was exceptionally small (i.e., in most cases $p = 10^{-10}$ or smaller).

Fixed effects	β ± se	z-value	p-value
Intercept	0.71 ± 0.010	69.2	**<0.001**
Lichen selection	0.091 ± 0.011	7.95	**<0.001**
Rocky barren selection	–0.0098 ± 0.010	–0.88	0.38
Wetland selection	0.042 ± 0.011	3.81	**<0.001**
Conifer selection	–0.014 ± 0.011	–1.23	0.21
Season (winter)[1]	–0.038 ± 0.007	–5.18	**<0.001**
Year[2]			
-2008	0.007 ± 0.010	0.69	0.48
-2009	–0.017 ± 0.010	–1.61	0.11
-2010	–0.034 ± 0.010	–3.25	**0.001**
-2011	–0.011 ± 0.012	–0.97	0.33
-2012	–0.030 ± 0.014	–2.10	**0.04**
-2013	–0.044 ± 0.020	–2.17	**0.03**
**Random effects**	**Variance ± SD**		
Herd:ID	0.0012 ± 0.035		
ID	0.0018 ± 0.043		
Residual	0.0066 ± 0.081		

[1]Reference category: calving.

[2]Reference category: 2007.

Table S4: Summary of resource selection models for caribou in winter and calving, including beta coefficients (± 95% confidence intervals) for four habitat types and elevation. Models included data for 127 individual caribou in both seasons.

	**Winter**	**Calving**
**Variable**	**Coefficient (± 95% confidence intervals)**	**Coefficient (± 95% confidence intervals)**
Intercept	–2.53 (–2.55, –2.51)	–2.36 (–2.38, –2.35)
Wetland	0.0005 (–85.5, 85.5)	0.0007 (–95.6, 95.6)
Conifer forest	–0.18 (–0.34, –0.03)	0.83 (0.72, 0.94)
Rocky barrens	0.32 (0.05, 0.60)	–0.14 (–0.49, 0.21)
Lichen barrens	1.77 (1.70, 1.83)	0.08 (–0.06, 0.24)
Elevation	–1.40 (–1.44, –1.35)	–0.89 (–0.93, –0.85)

Table S5: Estimates for fixed effects are given for the global model with social strength, habitat specialization, and reproductive success (fitness) as co-response variables in a tri-variate Bayesian mixed model testing the effects of population density, herd, year (2007–2013), and season (winter or calving). 95% credible intervals are displayed in brackets for each posterior mean. P-values <0.001 are noted if the estimated Bayesian p-value was exceptionally small (i.e., in most cases p = 10^{-10} or smaller) and significance is assessed based on whether 95% credible intervals overlap zero.

	**Social strength**		**Habitat specialization**		**Fitness**	
**Variable**	**Posterior Mean**	**MCMC p-value**	**Posterior Mean**	**MCMC p-value**	**Posterior Mean**	**MCMC p-value**
Social strength	–0.0001 (–0.01, 0.016)	0.98	–	–	–	–
Habitat specialization	–	–	0.02 (–0.06, 0.48)	0.11	–	–
Fitness	–	–	–	–	46.2 (–14.9, 125.2)	0.14
Density	–0.008 (–0.07, 0.06)	0.81	–0.17 (–0.25, –0.10)	**<0.001**	–1.32 (–15.7, 11.9)	0.85
Year[1]						
-2008	–0.11 (–0.23, 0.03)	0.11	–0.008 (–0.21, 0.18)	0.93	–9.13 (–50.7, 24.3)	0.59
-2009	–0.20 (–0.32, –0.08)	**0.001**	–0.30 (–0.49, –0.11)	**0.003**	21.5 (–15.9, 60.5)	0.22
-2010	–0.19 (–0.32, –0.63)	**0.003**	–0.45 (–0.65, –0.25)	**<0.001**	–17.5 (–57.8, 19.0)	0.32
-2011	–0.38 (–0.53, –0.23)	**<0.001**	–0.20 (–0.43, 0.01)	0.07	–5.8 (–46.8, 36.8)	0.76
-2012	–0.34 (–0.53, –0.15)	**<0.001**	–0.30 (–0.55, –0.05)	**0.01**	11.0 (–36.4, 64.1)	0.64
-2013	–0.56 (–0.81, –0.29)	**<0.001**	–0.61 (–1.01, –0.25)	**0.001**	–45.4 (–135.7, 21.3)	0.18
Herd[2]						
-Grey River	–0.39 (–0.56, –0.16)	0.005	–0.25 (–0.56, 0.07)	0.11	–39.8 (–132.8, 41.4)	0.31
-La Poile	0.007 (–0.19, 0.27)	0.95	0.16 (–0.15, 0.49)	0.33	–22.5 (–117.1, 52.9)	0.55
-Middle Ridge	0.13 (–0.75, 0.34)	0.19	0.19 (–0.09, 0.34)	0.18	20.4 (–53.7, 97.9)	0.47
-Pot Hill	–0.47 (–0.68, –0.26)	**<0.001**	–0.61 (–0.95, –0.28)	**<0.001**	–31.9 (–128.2, 48.9)	0.43
-Topsails	–0.13 (–0.35, 0.48)	0.17	0.06 (–0.23, 0.36)	0.67	1.79 (–76.8, 82.5)	0.97
Season						
-Winter[3]	0.06 (0.43, 0.73)	**<0.001**	0.003 (–0.09, 0.15)	0.65	1.45 (–20.1, 24.9)	0.89

[1] Reference category: 2007.
[2] Reference category: Buchans.
[3]Reference category: calving

Table S6: Estimates for fixed effects are given for the low-density model with habitat specialization and reproductive success (fitness) as co-response variables in a bi-variate Bayesian mixed model testing the effects of population density, herd, year (2007–2013), and season (winter or calving). 95% credible intervals are displayed in brackets for each posterior mean. P-values <0.001 are noted if the estimated Bayesian p-value was exceptionally small (i.e., in most cases p = 10^{-10} or smaller) and significance is assessed based on whether 95% credible intervals overlap zero.

	**Low density**			
	**Habitat specialization**		**Fitness**	
**Variable**	**Posterior Mean**	**MCMC p-value**	**Posterior Mean**	**MCMC p-value**
Habitat specialization	–1.97 (–3.61, –0.48)	**0.01**	–	–
Fitness	–	–	74.8 (–50.6, 664.4)	0.80
Social strength	14.4 (2.27, 26.3)	0.01	–481 (–4642, 3795)	0.84
Density	–1.75 (–3.44, 0.009)	**0.05**	–84.8 (–736, 611)	0.79
Year[1]				
-2008	–0.02 (–0.65, 0.60)	0.96	34.4 (–226.6, 261.5)	0.77
-2009	–0.53 (–1.04, 0.03)	0.05	50.8 (–161.8, 256.3)	0.63
-2010	–0.50 (–1.07, 0.05)	0.08	–28.9 (–248.6, 174.8)	0.76
-2011	–0.43 (–1.01, 0.18)	0.16	–52.8 (–276.1, 180.8)	0.63
-2012	–0.11 (0.90, 0.69)	0.80	33.2 (–293.8, 332.6)	0.84
-2013	–0.69 (–1.48, 0.05)	0.07	–110.5 (–391.6, 156.5)	0.42
Herd[2]				
-Grey River	0.15 (–0.50, 0.80)	0.66	–131 (–337.7, 87.7)	0.21
-La Poile	0.87 (–0.28, 1.99)	0.13	–158.9 (–585, 242)	0.44
-Middle Ridge	0.93 (0.26, 1.64)	**0.01**	–12.8 (–246.9, 222.6)	0.92
-Pot Hill	–0.39 (–1.05, 0.27)	0.25	–8.83 (–301, 121.7)	0.41
Season				
-Winter[3]	0.56 (0.76, 1.09)	**0.04**	–89.3 (–303.5, 118.6)	0.40

[1] Reference category: 2007.
[2] Reference category: Buchans.
[3]Reference category: calving

Table S7: Estimates for fixed effects are given for the high density model with habitat specialization and reproductive success (fitness) as co-response variables in a bi-variate Bayesian mixed model testing the effects of population density, herd, year (2007–2013), and season (winter or calving). 95% credible intervals are displayed in brackets for each posterior mean. P-values <0.001 are noted if the estimated Bayesian p-value was exceptionally small (i.e., in most cases p = 10^{-10} or smaller) and significance is assessed based on whether 95% credible intervals overlap zero.

	**High density**			
	**Habitat specialization**		**Fitness**	
**Variable**	**Posterior Mean**	**MCMC p-value**	**Posterior Mean**	**MCMC p-value**
Habitat specialization	–1.51 (–2.30, –0.67)	0.001	–	–
Fitness	–	–	111 (–166, 382.8)	0.43
Social strength	9.18 (–4.98, 2.24)	0.20	–1651 (6137, 3059)	0.48
Density	0.26 (–0.05, 0.54)	0.09	9.94 (–90.6, 109.1)	0.84
Year[1]				
-2008	1.05 (0.42, 1.59)	**<0.001**	–18.5 (–211.1, 164.2)	0.85
-2009	0.22 (–0.45, 0.89)	0.52	136.6 (–98.8, 364.2)	0.23
-2010	1.51 (0.60, 2.43)	**0.001**	–162.5 (–453.6, 152.9)	0.27
-2011	2.23 (1.42, 2.99)	**<0.001**	–88.2 (–336.4, 152.1)	0.47
-2012	1.67 (0.98, 2.35)	**<0.001**	1.64 (–217.5, 216.9)	0.98
-2013	0.39 (–0.62, 1.53)	0.48	33.4 (–315.2, 413.3)	0.87
Herd[2]				
-Grey River	–1.56 (–2.41, –0.69)	**0.0005**	–65.9 (–342.6, 229)	0.64
-La Poile	0.11 (–0.46, 0.62)	0.69	–8.9 (–265.2, 107.7)	0.34
-Middle Ridge	–0.31 (–1.20, 0.59)	0.51	–109.6 (–440, 181)	0.47
-Pot Hill	0.52 (–0.21, 1.25)	0.17	–119.7 (–382.2, 136)	0.35
-Topsails	–0.98 (–1.83, –0.14)	**0.02**	144.3 (–150.3, 408.4)	0.29
Season				
-Winter[3]	0.91 (0.51, 1.23)	**<0.001**	15.8 (–121.3, 137.8)	0.80

[1] Reference category: 2007.
[2] Reference category: Buchans.
[3]Reference category: calving

Table S8: Estimates for fixed effects are given for the low density model with social strength and reproductive success (fitness) as co-response variables in a bi-variate Bayesian mixed model testing the effects of population density, herd, year (2007–2013), and season (winter or calving). 95% credible intervals are displayed in brackets for each posterior mean. P-values <0.001 are noted if the estimated Bayesian p-value was exceptionally small (i.e., in most cases p = 10^{-10} or smaller) and significance is assessed based on whether 95% credible intervals overlap zero.

	**Low density**			
	**Social strength**		**Fitness**	
**Variable**	**Posterior Mean**	**MCMC p-value**	**Posterior Mean**	**MCMC p-value**
Social strength	–2.03 (–3.98, –0.07)	**0.04**	–	–
Fitness	–	–	144.2 (–478.7, 772.3)	0.64
Habitat specialization	1.82 (0.44, 3.27)	**0.01**	–84.6 (–541.8, 337.6)	0.70
Density	–1.01 (–3.11, 1.10)	0.34	–72.0 (–715.8, 631.6)	0.83
Year[1]				
-2008	0.23 (–0.57, 0.96)	0.56	28.2 (–195.7, 276.2)	0.80
-2009	–0.54 (–1.17, 0.13)	0.11	47.9 (–162.1, 249.2)	0.66
-2010	–0.54 (–1.18, 0.13)	0.12	–32.5 (–239.65, 179.6)	0.75
-2011	–0.54 (–1.25, 0.20)	0.15	–54.5 (–280.6, 180.8)	0.63
-2012	–0.85 (–1.90, 0.14)	0.10	22.5 (–275.8, 340.5)	0.88
-2013	–0.60 (–1.47, 0.30)	0.19	–115.9 (–411.5, 151.6)	0.41
Herd[2]				
-Grey River	–0.71 (–1.34, –0.03)	0.03	–128.7 (–342.2, 97.4)	0.24
-La Poile	–0.38 (–1.67, 0.99)	0.57	–142.4 (–554.6, 274.7)	0.51
-Middle Ridge	0.19 (–0.54, 1.02)	0.62	–7.91 (–249.4, 230.8)	0.96
-Pot Hill	–0.22 (–0.96, 0.47)	0.55	–84.7 (–318.4, 128.4)	0.46
Season				
-Winter[3]	0.64 (–0.03, 1.33)	0.06	–88.3 (–293.6, 126.2)	0.39

[1] Reference category: 2007.
[2] Reference category: Buchans.
[3]Reference category: calving

Table S9: Estimates for fixed effects are given for the high density model with social strength and reproductive success (fitness) as co-response variables in a bi-variate Bayesian mixed model testing the effects of population density, herd, year (2007–2013), and season (winter or calving). 95% credible intervals are displayed in brackets for each posterior mean. P-values <0.001 are noted if the estimated Bayesian p-value was exceptionally small (i.e., in most cases $p = 10^{-10}$ or smaller) and significance is assessed based on whether 95% credible intervals overlap zero.

	**High density**			
	**Social strength**		**Fitness**	
**Variable**	**Posterior Mean**	**MCMC p-value**	**Posterior Mean**	**MCMC p-value**
Social strength	0.26 (–0.91, 1.44)	0.67	–	–
Fitness	–	–	231.8 (–146.8, 627.7)	0.23
Habitat specialization	0.80 (–0.68, 2.28)	0.31	–250.7 (–767.9, 274.5)	0.34
Density	–0.03 (–0.35, 0.27)	0.85	14.8 (–89.4, 117.3)	0.77
Year[1]				
-2008	–1.00 (–1.59, –0.41)	**<0.001**	25.9 (–178.1, 210.6)	0.77
-2009	–0.71 (–5.66, –0.007)	**0.04**	159.0 (–56.6, 408.7)	0.15
-2010	–0.79 (–1.75, 0.18)	0.11	–126.4 (–459.3, 169.6)	0.41
-2011	–0.77 (–1.65, 0.09)	0.07	–23.3 (–300.8, 261.8)	0.86
-2012	–0.97 (–1.75, –0.26)	**0.01**	57.1 (–167.7, 292.3)	0.64
-2013	–1.43 (–2.51, –0.26)	**0.02**	7.16 (–287.7, 446.4)	0.69
Herd[2]				
-Grey River	–0.65 (–1.49, 0.32)	0.16	–109.1 (–409, 180.4)	0.45
-La Poile	–0.25 (–0.83, 0.29)	0.38	–86.6 (–271.1, 89.6)	0.35
-Middle Ridge	0.20 (–0.81, 1.15)	0.68	–128.5 (–437.2, 186.9)	0.41
-Pot Hill	–0.99 (–1.71, –0.29)	**0.008**	–97.9 (–352.3, 150.1)	0.44
-Topsails	–0.59 (–1.49, 0.29)	0.19	114.3 (–153.2, 404.3)	0.42
Season				
-Winter[3]	0.29 (–0.12, 0.72)	0.18	33.9 (–100.9, 171.7)	0.64

[1] Reference category: 2007.

[2] Reference category: Buchans.

[3] Reference category: calving

REFERENCES

Bastille-Rousseau, G., Schaefer, J.A., Lewis, K.P., Mumma, M.A., Ellington, E.H., Rayl, N.D., *et al.* (2016). Phase-dependent climate-predator interactions explain three decades of variation in neonatal caribou survival. *Journal of Animal Ecology*, 85, 445–456.

Bastille-Rousseau, G., Schaefer, J.A., Mahoney, S.P. & Murray, D.L. (2013a). Population decline in semi-migratory caribou (*Rangifer tarandus*): intrinsic or extrinsic drivers? *Canadian Journal of Zoology*, 91, 820–828.

Bastille-Rousseau, G., Schaefer, J.A., Mahoney, S.P. & Murray, D.L. (2013b). Population decline in semi-migratory caribou (*Rangifer tarandus*): intrinsic or extrinsic drivers? *Canadian Journal of Zoology*, 91, 820–828.

Bell, A.M., Hankison, S.J. & Laskowski, K.L. (2009). The repeatability of behaviour: a meta-analysis. *Animal Behaviour*, 77, 771–783.

Bjørneraas, K., Van Moorter, B., Rolandsen, C.M. & Herfindal, I. (2010). Screening global positioning system location data for errors using animal movement characteristics. *Journal of Wildlife Management*, 74, 1361–1366.

Bolnick, D.I., Svanbäck, R., Fordyce, J.A., Yang, L.H., Davis, J.M., Hulsey, C.D., *et al.* (2003). The ecology of individuals: Incidence and implications of individual specialization. *The American Naturalist*, 161, 1–28.

Bolnick, D.I., Yang, L.H., Fordyce, J.A., Davis, J.M. & Svanbäck, R. (2002). Measuring individual-level resource specialization. *Ecology*, 83, 2936–2941.

Brooks, M.E., Kristensen, K., van Benthem, K.J., Magnusson, A., Berg, C.W., Nielsen, A., *et al.* (2017). glmmTMB balances speed and flexibility among packages for zero-inflated generalized linear mixed modeling. *R Journal*, 9, 378–400.

Cairns, S.J. & Schwager, S.J. (1987). A comparison of association indices. *Animal Behaviour*, 35, 1454–1469.

Carlson, B.S., Rotics, S., Nathan, R., Wikelski, M. & Getz, W. (2021). Individual environmental niches in mobile organisms. *Nature Communications*, 12, 4572.

Csárdi, G. & Nepusz, T. (2006). The igraph software package for complex network research. *InterJournal Complex Systems*, 1695, 1–9.

Dingemanse, N.J., Kazem, A.J.N., Réale, D. & Wright, J. (2010). Behavioural reaction norms: animal personality meets individual plasticity. *Trends in Ecology and Evolution*, 25, 81–89.

Ellington, E.H., Lewis, K.P., Koen, E.L. & Vander Wal, E. (2020). Divergent estimates of herd-wide caribou calf survival: Ecological factors and methodological biases. *Ecology & Evolution*, 1–20.

Farine, D.R. (2014). Measuring phenotypic assortment in animal social networks: Weighted associations are more robust than binary edges. *Animal Behaviour*, 89, 141–153.

Farine, D.R. (2017). A guide to null models for animal social network analysis. *Methods in Ecology and Evolution*, 8, 1309–1320.

Farine, D.R. & Whitehead, H. (2015). Constructing, conducting and interpreting animal social network analysis. *Journal of Animal Ecology*, 84, 1144–1163.

Festa-Bianchet, M., Gaillard, J.-M. & Jorgenson, J.T. (1998). Mass- and density-dependent reproductive success and reproductive costs in a capital breeder. *American Naturalist*, 152, 367–379.

Fortin, D., Morris, D.W. & McLouglin, P.D. (2008). Habitat selection and the evolution of specialists in heterogeneous environments. *Israel Journal of Ecology & Evolution*, 54, 295–309.

Gaillard, J.-M., Festa-bianchet, M., Delorme, D. & Jorgenson, J. (2000). Body mass and individual fitness in female ungulates: bigger is not always better. *Proceedings of the Royal Society B*, 471–477.

Gunn, A. (2003). Voles, lemmings and caribou - population cycles revisited? *Rangifer*, 23, 105.

Hadfield, J.D. (2010). MCMC methods for multi-respoinse generalized linear mixed models: The MCMCglmm R package. *Journal of Statistical Software*, 33, 1–22.

Harris, N.C., Kauffman, M.J. & Mills, L.S. (2010). Inferences About Ungulate Population Dynamics Derived From Age Ratios. *Journal of Wildlife Management*, 72, 1143–1151.

Johnson, D.H. (1980). The comparison of usage and availability measurements for evaluating resource preference. *Ecology*, 61, 65–71.

Mahoney, S.P. & Virgl, J.A. (2003). Habitat selection and demography of a nonmigratory woodland caribou population in Newfoundland. *Canadian Journal of Zoology*, 81, 321–334.

McLoughlin, P.D., Boyce, M.S., Coulson, T. & Clutton-Brock, T. (2006). Lifetime reproductive success and density-dependent, multi-variable resource selection. *Proceedings of the Royal Society B*, 273, 1449–1454.

Muff, S., Signer, J. & Fieberg, J. (2020). Accounting for individual-specific variation in habitat-selection studies: Efficient estimation of mixed-effects models using Bayesian or frequentist computation. *Journal of Animal Ecology*, 89, 80–92.

Nakagawa, S. & Schielzeth, H. (2013). A general and simple method for obtaining R2 from generalized linear mixed-effects models. *Methods in Ecology and Evolution*, 4, 133–142.

Northrup, J.M., Vander Wal, E., Bonar, M., Fieberg, J., Laforge, M.P., Leclerc, M., *et al.* (2022). Conceptual and methodological advances in habitat-selection modeling: guidelines for ecology and evolution. *Ecological Applications*, 32, 1–31.

Robitaille, A.L., Webber, Q.M.R. & Vander Wal, E. (2019). Conducting social network analysis with animal telemetry data: applications and methods using spatsoc. *Methods in Ecology and Evolution*, 10, 1203–1211.

Schaefer, J.A., Mahoney, S.P., Weir, J.N., Luther, J.G. & Soulliere, C.E. (2016). Decades of habitat use reveal food limitation of Newfoundland caribou. *Journal of Mammalogy*, 97, 386–393.

Sikes, R.S. & Mammalogists, A.C. and U.C. of the A.S.O. (2016). 2016 Guidelines of the American Society of Mammalogists for the use of wild mammals in research and education. *Journal of Mammalogy*, 97, 663–688.

Spiegel, O., Leu, S.T., Sih, A. & Bull, C.M. (2016). Socially interacting or indifferent neighbours? Randomization of movement paths to tease apart social preference and spatial constraints. *Methods in Ecology and Evolution*, 7, 971–979.

Street, G.M., Potts, J.R., Börger, L., Beasley, J.C., Demarais, S., Fryxell, J.M., *et al.* (2021). Solving the sample size problem for resource selection functions. *Methods in Ecology and Evolution*, 2021, 1–11.

Tinker, M.T., Bentall, G. & Estes, J.A. (2008). Food limitation leads to behavioral diversification and dietary specialization in sea otters. *Proceedings of the National Academy of Sciences of the United States of America*, 105, 560–565.

Virgl, J.A., Rettie, W.J. & Coulton, D.W. (2017). Spatial and temporal changes in seasonal range attributes in a declining barren-ground caribou herd. *Rangifer*, 37, 31.

Webber, Q.M.R. & Vander Wal, E. (2018). An evolutionary framework outlining the integration of individual social and spatial ecology. *Journal of Animal Ecology*, 87, 113–127.

Webber, Q.M.R. & Vander Wal, E. (2021). Context-dependent group size: effects of population density, habitat, and season. *Behavioral Ecology*, 1–12.

Weir, J.N., Morrison, S.F., Luther, J.G. & Mahoney, S.P. (2014). *Status of the Newfoundland population of woodland caribou*.

Zaccarelli, N., Bolnick, D.I. & Mancinelli, G. (2013). RInSp: an R package for the analysis of individual specialization in resource use. *Methods in Ecology and Evolution*, 4, 1018–1023.
